# Histone demethylase KDM6B promotes postnatal oligodendrocyte maturation and cortical myelination

**DOI:** 10.3389/fncel.2026.1832157

**Published:** 2026-05-19

**Authors:** Ruth Lambries, Zihan Shen, George I. Mias, Jin He

**Affiliations:** 1 Department of Biochemistry and Molecular Biology, College of Natural Science, Michigan State University, East Lansing, MI, United States; 2 Institute for Quantitative Health Science and Engineering, Michigan State University, East Lansing, MI, United States

**Keywords:** histone demethylase, KDM6B, myelination, neurodevelopment, oligodendrocyte

## Abstract

**Introduction:**

Postnatal cortical myelination requires epigenetic activation of oligodendrocyte gene programs, but the role of histone demethylases *in vivo* remains unclear.

**Methods:**

We conditionally deleted *Kdm6b* in Emx1^+^ dorsal telencephalic progenitors and analyzed oligodendrocyte lineage progression, performed lineage-specific RNA-seq, assessed H3K27me3 occupancy by chromatin immunoprecipitation, and tested functional rescue via *Sox10* induction.

**Results:**

*Kdm6b* deletion delayed cortical myelination and impaired oligodendrocyte maturation without affecting precursor density. Pre-myelinating and mature oligodendrocyte markers were reduced. RNA-seq revealed early suppression of *Sox10*, followed by downregulation of myelin genes. Consistently, H3K27me3 enrichment increased at *Sox10* regulatory regions. *Sox10* induction partially rescued myelination deficits.

**Discussion:**

These findings define a KDM6B→*SOX10* axis that promotes oligodendrocyte maturation and cortical myelination by restricting repressive H3K27me3, providing mechanistic insight into epigenetic control of myelination with relevance to KDM6B-associated neurodevelopmental disorders.

## Introduction

Myelination is a critical feature of postnatal brain maturation that supports rapid saltatory conduction and metabolic coupling between axons and glia (Fletcher et al., 2021; Zhang et al., 2025). In the neocortex, oligodendrocyte lineage progression-from progenitors to myelinating oligodendrocytes-unfolds predominantly postnatally and is tightly coordinated with circuit assembly and activity (Kuhn et al., 2019). This process is controlled by transcriptional programs that drive differentiation and myelin gene expression. Among these regulators, SOX10 is a central and essential factor that promotes oligodendrocyte maturation and activates genes required for myelin sheath formation and stability (Pozniak et al., 2010; Elbaz and Popko, 2019). However, the epigenetic mechanisms that enable timely activation of the SOX10-dependent myelin program in the developing cortex remain incompletely defined (Liu et al., 2016).

A major layer of epigenetic control involves covalent histone modifications that shape chromatin accessibility and gene expression. Trimethylation of histone H3 lysine 27 (H3K27me3), deposited by the Polycomb Repressive Complex 2 (PRC2), is a canonical repressive mark that constrains developmental competence (Cao et al., 2002; Mozgova et al., 2015). The histone demethylase KDM6B catalyzes removal of H3K27me3, thereby antagonizing PRC2 and facilitating transcriptional activation at developmental loci (Agger et al., 2007; Sen et al., 2008). KDM6B has been implicated in multiple neural processes, including neural commitment, lineage specification, postnatal neurogenesis, neuronal survival, and neuronal maturation (Burgold et al., 2008; Park et al., 2014; Wijayatunge et al., 2014, 2018; Shan et al., 2020). These findings suggest that KDM6B may also regulate postnatal oligodendrocyte maturation and cortical myelination by relieving repressive chromatin at key lineage regulators.

The clinical relevance of KDM6B is underscored by human genetics. Recent large-scale studies identify KDM6B as a high-confidence autism spectrum disorder (ASD) risk gene, and multiple case reports describe children with disruptive or missense KDM6B variants presenting with neurodevelopmental phenotypes that include intellectual disability, delayed language/motor development, and attention-deficit/hyperactivity disorder (ADHD) behaviors (Stolerman et al., 2019; Satterstrom et al., 2020; Insa Pineda and Gómez González, 2021; Rots et al., 2023, 2025). Together with experimental evidence that heterozygous loss of Kdm6b in mice produces ASD- and ADHD-like behaviors, these findings suggest that KDM6B-dependent chromatin regulation contributes to brain development and function (Gao et al., 2022). Whether defects in oligodendrocyte maturation and myelination participate in the pathophysiology of KDM6B-related neurodevelopmental disorders, however, remains unknown.

In the present study, we examined the role of KDM6B in postnatal oligodendrocyte maturation and cortical myelination. We conditionally deleted *Kdm6b* in Emx1-positive dorsal telencephalic progenitors, a lineage that contributes to cortical glutamatergic neurons, astrocytes, and a subset of oligodendrocytes. We combined postnatal histological analysis with stage-specific oligodendrocyte markers, lineage-specific RNA-seq, and chromatin immunoprecipitation analysis of H3K27me3. To test functional causality, we further used temporally controlled, Tet-inducible *Sox10* expression *in vivo*.

We show that loss of Kdm6b delays postnatal cortical myelination and impairs oligodendrocyte maturation without detectably altering postnatal OPC density. At the molecular level, Sox10 is reduced early, followed by broader downregulation of myelin-associated genes. At the chromatin level, Kdm6b-deficient Emx1-lineage cells show increased H3K27me3 at promoter-proximal regions of Sox10. Importantly, temporally controlled Sox10 induction partially rescues cortical myelination in Kdm6b-cKO mice. Together, these findings identify a KDM6B→SOX10 regulatory axis that promotes postnatal oligodendrocyte maturation and cortical myelination in the developing cortex.

## Materials and methods

### Mice

Founder conditional *Kdm6b* knockout (*Kdm6b*-cKO) mice were generated by *in vitro* fertilization at the Michigan State University (MSU) Transgenic Core, as reported in our previous study (Gao et al., 2022). Mice were housed under standard conditions (12 h light/12 h dark cycle) with *ad libitum* access to food and water. All procedures were approved by the Michigan State University Institutional Animal Care and Use Committee (IACUC, PROTO202300162) and conducted in accordance with institutional guidelines.

### Mouse breeding strategy

All mice were backcrossed to C57BL/6 for more than five generations to achieve a congenic C57BL/6 background. Conditional knockouts and controls were generated by crossing Emx1-Cre mice [B6.129S2-Emx1^TM1^(cre)^Krj^/J, The Jackson Laboratory] with *Kdm6b* floxed mice. Littermates included wild-type (*Kdm6b*^wt/wt^) and conditional knockout (Emx1-Cre; Kdm6b^f/f^) animals. For breeding, *Kdm6b*^wt/f^ × *Kdm6b*^wt/f^ matings produced *Kdm6b*^wt/wt^, *Kdm6b*^wt/f^, and *Kdm6b*^f/f^ offspring. All lines were maintained on a SUN1-GFP reporter background [B6;129-*Gt(ROSA)26Sor^TM 5(CAG–Sun1/sfGFP)Nat^*/J, The Jackson Laboratory].

### In uterus electroporation

E15.5 dams were anesthetized with isoflurane on a 37 °C heated stage, the abdomen was aseptically prepared, and a midline laparotomy was performed to externalize the uterine horns on warm, saline-moistened gauze. Endotoxin-free plasmids (1 μg/μL total in sterile PBS with 0.01% Fast Green) were loaded into a pulled glass capillary attached to a mouth pipette and 1.0 μL DNA was injected into the lateral ventricle. Tweezer-type platinum electrodes were positioned to direct the anode toward the dorsal pallium and 5 square-wave pulses were delivered with a pulse generator (40 V, 50 ms width, 950 ms interval). After electroporation, uterine horns were returned to the abdominal cavity, the peritoneum and skin were closed with absorbable sutures and wound clips, and dams received carprofen per IACUC guidelines. The procedure was approved by the Michigan State University Institutional Animal Care and Use Committee (IACUC) and conducted in accordance with institutional guidelines.

### Doxycycline administration

To induce gene expression in offspring carrying a doxycycline-inducible transgene, pregnant dams were provided with doxycycline-containing drinking water starting at embryonic day 18.5 (E18.5) and maintained continuously until postnatal day 21 (P16). The doxycycline solution was prepared fresh every 2 days by dissolving doxycycline hyclate (Sigma-Aldrich) at a final concentration of 2 mg/mL in 2% sucrose (w/v) to improve palatability and protected from light using amber bottles. Dams were housed under standard conditions with *ad libitum* access to the doxycycline water, which ensured sustained systemic exposure through lactation to activate the tetracycline-responsive transgene in the pups during late embryonic and early postnatal development. The procedure was approved by the Michigan State University Institutional Animal Care and Use Committee (IACUC) and conducted in accordance with institutional guidelines.

### Tissue processing for histology

P16, P24, and P35 mice were anesthetized by avertin and transcardially perfused with ice-cold phosphate-buffered saline followed by 4% paraformaldehyde (PFA) in PBS. Brains were dissected and post-fixed in 4% PFA at 4 °C overnight, then cryoprotected in 30% sucrose in PBS at 4 °C until tissue sank. Cryoprotected brains were embedded in optimal cutting temperature (OCT) compound in molds, frozen on dry-ice or a pre-chilled isopentane bath, and stored at −80 °C. Coronal sections were cut at 10 μm on a cryostat (Leica CM1950).

### Immunostaining

Slide-mounted OCT cryosections were rinsed in PBS and permeabilized/blocked for 1 h at RT in PBS containing 0.3% Triton X-100 and 5% normal donkey. Rabbit anti-MBP (Cell Signaling), Rabbit anti-SOX10 (Cell Signaling), Rabbit anti-MAG (Cell Signaling), Rabbit anti-MOG (Cell Signaling), Rabbit anti-NeuN (Cell Signaling), and Rabbit anti-BCAS1 (Synaptic Systems) diluted in the same buffer (1:400) were applied overnight at 4 °C in a humidified chamber. The next day, sections were washed in PBS and incubated with Donkey anti-rabbit Alexa Fluor 680–conjugated secondary antibodies (1:400, Jackson ImmunoResearch) for 1 h at RT protected from light, followed by PBS washes. Nuclei were counterstained with DAPI (0.5 μg/mL, 5 min), briefly rinsed, and coverslipped with antifade mounting medium.

### Imaging

Images were acquired using a ZEISS Axio Observer Z1 inverted fluorescence microscope equipped with 2.5× and 10× objective lenses and captured with ZEN Pro software (ZEISS).

### Imaging data analysis

Mean fluorescence intensity (MFI) of MBP immunofluorescence was quantified using ImageJ (NIH). All images were converted to 8-bit format to standardize intensity scaling across samples. One image per sample was analyzed. A region of interest (ROI) encompassing a high-density myelinated area immediately above the corpus callosum was used as a reference region and set as 100 for normalization. The sampling ROI encompassed the outer extent of cortical myelinated axons and was used as the primary measurement region for MFI quantification. For the MAG, MOG, and MBP fiber length proportion analysis, the same sections used for MFI analysis were used to quantify cortical myelination length using ImageJ. Total cortical width was measured by obtaining five independent linear measurements (in pixels) from the top of the corpus callosum to the pial surface within the superior lateral cortex. These measurements were averaged to yield a mean cortical width for each sample. The extent of myelination was determined by taking five measurements from the top of the corpus callosum to the distal boundary of the myelinated region. These values were averaged to obtain the mean myelinated length. The proportion of cortical myelination was calculated by dividing the mean myelinated length by the mean cortical width for each sample. This analysis was performed for all samples used in the study. SOX10, PDGFRA, and NeuN immunostaining was quantified using ImageJ. All images were converted to 8-bit format to allow automated particle analysis. For each sample, three 400 × 400-pixel ROIs were randomly selected within the analyzed hemisphere. Automated particle counting was performed for each ROI using ImageJ’s particle analysis function. Cellular density was calculated by dividing the particle count by the ROI area (160,000 pixels^2^). Density values from the three ROIs were averaged to obtain a single value per sample for statistical analysis.

### Fluorescence-activated nuclei sorting

Mouse cortices were rapidly dissected on ice. Nuclei were isolated sucrose gradient centrifugation. Briefly, 14 mL of sucrose cushion (1.8 M sucrose, 10 mM Tris-HCl pH 8.0, 3 mM MgCl2) was added to the bottom of Beckman Coulter ultracentrifuge tubes. Using a glass Dounce homogenizer, a freshly isolated or frozen cortex was homogenized in 12 mL homogenization buffer (0.32 M sucrose, 5 mM CaCl2 3 mM MgCl2, 10 mM Tris-HCl pH 8.0, 0.1% Triton X-100, 0.1 mM EDTA with strokes of the loose pestle followed by strokes of the tight pestle. Homogenates (∼12 mL) were carefully layered onto the sucrose cushion, and 10 mL homogenization buffer was added on top. Gradients were centrifuged at 25,000 rpm for 2.5 h at 4 °C in a Beckman Coulter L8-70M ultracentrifuge using an SW28 swinging-bucket rotor. Supernatants were removed by aspiration. Nuclei were resuspended in 0.01% BSA in DPBS passed through a 40-μm cell strainer. The SUN1-GFP + nuclei were sorted by S3e sorter (Bio-Rad).

### qRT-PCR assays

Total RNA was extracted using QIAshredder and RNeasy spin columns (QIAGEN) according to the manufacturer’s instructions. RNA was reverse-transcribed with HIScript Reverse Transcriptase III (Vazyme). Quantitative PCR was performed using SupRealQ Ultra Hunter SYBR qPCR Master Mix (U+) (Vazyme) on a CFX384 Touch Real-Time PCR Detection System (Bio-Rad). Primer sequences are listed in Supplementary Table 1.

### RNA-seq sample preparation for AVITI sequencing

Total RNA was extracted according to the manufacturer’s instructions. RNA-seq libraries were prepared from total RNA using the VAHTS Universal V10 RNA-seq Library Prep Kit for Illumina (Vazyme). Adapter-ligated cDNA was PCR-amplified and size-selected magnetic beads. DNA was measured by an Invitrogen Qubit fluorometer. Libraries were sequenced on NovaSeq6000 or Element AVITI sequencers at the Michigan State University Genomics Core.

### RNA-seq data analysis

RNA-seq analysis was performed essentially as described previously (Aljazi et al., 2021; Gao et al., 2021). Reads were aligned to the mouse reference genome (UCSC mm9) using HISAT2 (Kim et al., 2019). Gene-level abundance was normalized as reads per kilobase per million mapped reads (RPKM). Differential expression was assessed with Cuffdiff (Trapnell et al., 2012), using a false-discovery rate threshold of q < 0.05 and a > 2-fold change in RPKM between conditions. Differentially expressed gene sets were submitted to g:Profiler^1^ for Gene Ontology enrichment analysis.

### ChIP-qPCR analysis

Chromatin immunoprecipitation was performed as described previously (He et al., 2013). FANS-sorted nuclei (2–3 × 10^5^ per IP) were lysed in 1% SDS, 10 mM EDTA, 50 mM Tris-HCl (pH 8.0) supplemented with protease inhibitors. Chromatin was sonicated on a Covaris S220 to an average fragment size of ∼200–400 bp, clarified by centrifugation, and diluted 10× with ChIP dilution buffer (0.01% SDS, 1.1% Triton X-100, 1.2 mM EDTA, 16.7 mM Tris-HCl pH 8.0, 167 mM NaCl). Supernatants were incubated overnight at 4 °C with 2 μL rabbit polyclonal anti-H3K27me3 (Cell Signaling Technology; catalog and lot as in Antibody Table). Immune complexes were captured with Protein G Plus/Protein A magnetic beads (EMD Millipore). Beads were washed sequentially in low-salt, high-salt, LiCl, and TE buffers, eluted in 1% SDS/0.1 M NaHCO3, and crosslinks were reversed (65 °C, ≥4 h). DNA was purified by phenol–chloroform extraction and ethanol precipitation and used for library preparation. Quantitative PCR was performed using SupRealQ Ultra Hunter SYBR qPCR Master Mix (U+) (Vazyme) on a CFX384 Touch Real-Time PCR Detection System (Bio-Rad). Primer sequences are listed in Supplementary Table 1.

### Statistical analysis

All statistical analyses were performed using GraphPad Prism 8 (GraphPad Software). For comparisons between two groups, statistical significance was assessed using two-tailed unpaired Student’s *t*-tests. For comparisons involving multiple samples, group multiple-row *t*-tests were applied with Holm–Šidák correction for multiple comparisons. *P*-values < 0.05 were considered statistically significant. Data are presented as mean ± SEM.

## Results

### Loss of *Kdm6b* delays postnatal cortical myelination

**FIGURE 1:**
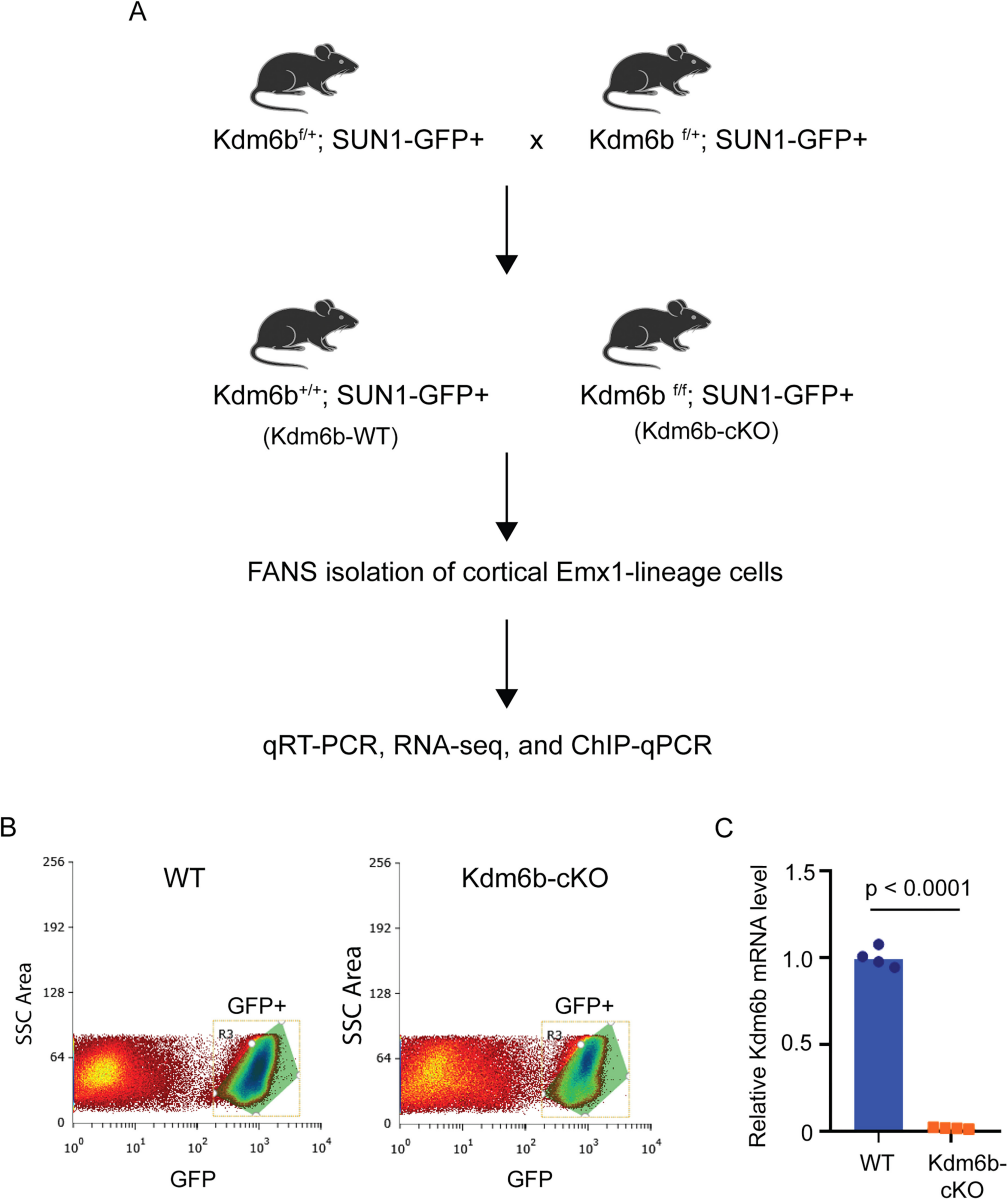
Generation and validation of *Kdm6b* conditional knockout in Emx1-lineage cortical cells. **(A)** Breeding scheme used to generate *Kdm6b*^+/+^; SUN1-GFP^+^ (WT) and Kdm6b^f/f^; SUN1-GFP^+^ (cKO) mice. Emx1-lineage cortical nuclei were isolated by fluorescence-activated nuclei sorting (FANS) based on GFP expression for downstream analyses. **(B)** Representative FANS plots showing gating of GFP^+^ cortical nuclei from WT and cKO mice. **(C)** qRT-PCR analysis of GFP^+^ nuclei confirming efficient loss of *Kdm6b* mRNA in cKO samples relative to WT controls. *Kdm6b* expression was normalized to *Gapdh*. Each dot represents one biological replicate.

To examine the role of KDM6B in cortical neural development, we conditionally deleted *Kdm6b* in Emx1***-***positive dorsal telencephalic progenitors, which give rise to cortical glutamatergic neurons, astrocytes, and oligodendrocytes, by crossing *Kdm6b*^flox/flox^ mice with the Emx1-Cre line (Gorski et al., 2002). Both wild-type (WT) and *Kdm6b***-**cKO cohorts were crossed to a SUN1-GFP reporter line to label Emx1**^+^** progeny, enabling lineage tracing and FANS (Fluorescence-Activated Nuclei Sorting)-based isolation of Emx1-lineage cells from cortices for downstream characterization (Figure 1A). To confirm knockout efficiency, we sorted SUN1-GFP^+^ cortical nuclei and performed quantitative real-time PCR (qRT-PCR) to measure *Kdm6b* mRNA. Relative to WT, *Kdm6b***-**cKO samples showed a > 95% reduction in *Kdm6b* transcript levels (i.e., <5% of WT), confirming efficient gene deletion (Figures 1B,C).

To determine whether loss of Kdm6b causes gross developmental abnormalities, we first measured body and brain weights at postnatal day (P)1 and P30. Body weights were comparable between WT and Kdm6b-cKO mice in both males and females at P1 and P30. Likewise, gross brain morphology and brain weight were not detectably altered in Kdm6b-cKO mice at either age (Supplementary Figure 1), indicating that Kdm6b deletion in the Emx1 lineage does not produce overt changes in overall postnatal growth or brain size.

**FIGURE 2:**
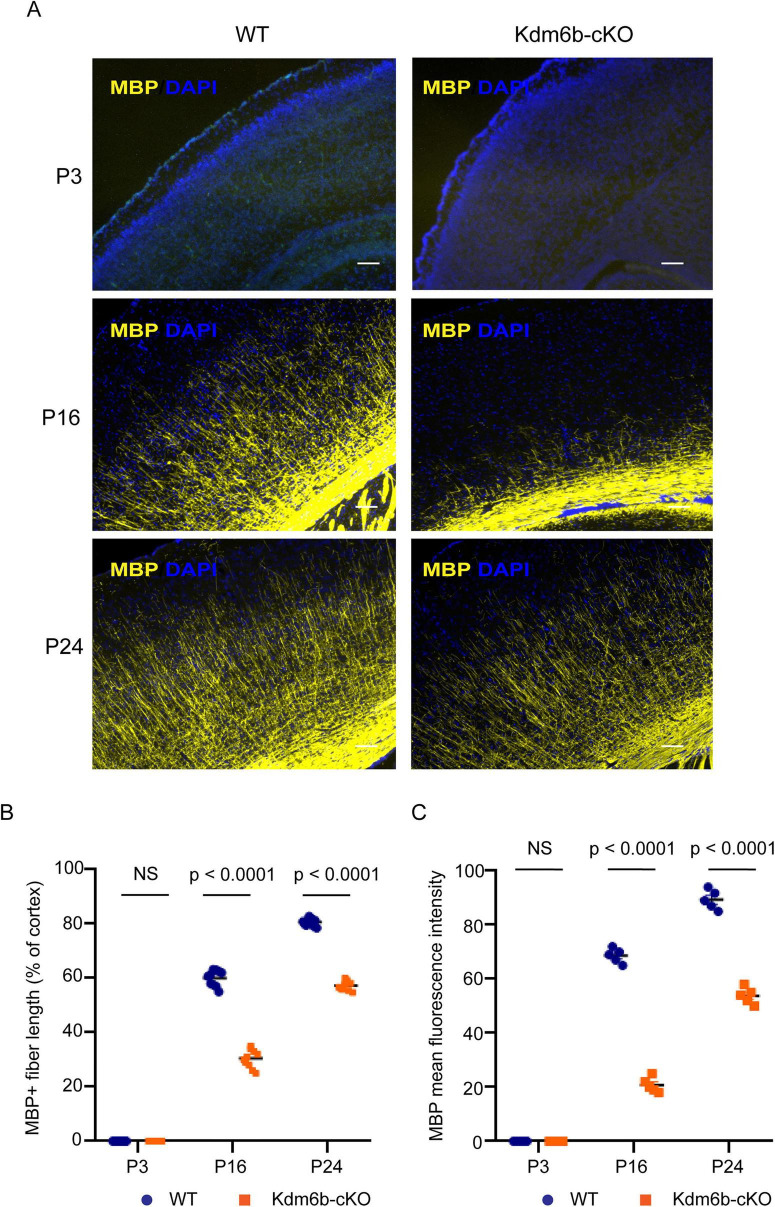
Delayed cortical myelination following *Kdm6b* deletion in Emx1-lineage cells. **(A)** Representative immunofluorescence images of cortical sections from *Kdm6b*-WT and *Kdm6b-cKO* mice at postnatal day 3 (P3), P16, and P24, stained for myelin basic protein (MBP, yellow) and DAPI (blue). WT cortices show robust, age-dependent increases in MBP-positive myelinated fibers, whereas *Kdm6b*-cKO cortices exhibit markedly reduced MBP labeling at P16 and P24. Scale bars, 200 μm. **(B)** Quantification of MBP-positive fiber length normalized to cortical length at P3, P16, and P24. No significant difference is observed at P3, whereas MBP-positive fiber length is significantly reduced in *Kdm6b*-cKO cortices at P16 and P24. Each dot represents an individual animal. Statistical significance is indicated (NS, not significant; *P* < 0.0001). **(C)** Quantification of mean MBP fluorescence intensity (MFI) at P3, P16, and P24. No significant difference is observed at P3, whereas MFI is significantly reduced in *Kdm6b*-cKO cortices at P16 and P24. Each dot represents an individual animal. Statistical significance is indicated (NS, not significant; *P* < 0.0001).

To assess postnatal cortical myelination, we performed immunostaining for myelin basic protein (MBP) at postnatal day (P) 3, P16, and P24. As expected, wild-type (WT) mice showed a progressive increase in MBP signal, with minimal or absent staining at P3, moderate staining at P16, and robust staining at P24. *Kdm6b*-cKO littermates followed a similar temporal trajectory but displayed markedly reduced MBP-positive neural fiber length and immunofluorescence intensity at P16 and P24 compared with WT (Figure 2), indicating that loss of *Kdm6b* in cortical neural progenitors impairs oligodendrocyte maturation and delays postnatal cortical myelination. To determine whether this deficit persists beyond P30, we further examined MBP staining at P35. MBP-positive fiber length remained significantly reduced in *Kdm6b*-cKO cortex at P35 (Supplementary Figure 2), indicating that although myelination progresses over time, the deficit persists into later postnatal stages.

### Loss of *Kdm6b* does not alter postnatal OPC density but disrupts oligodendrocyte maturation

**FIGURE 3:**
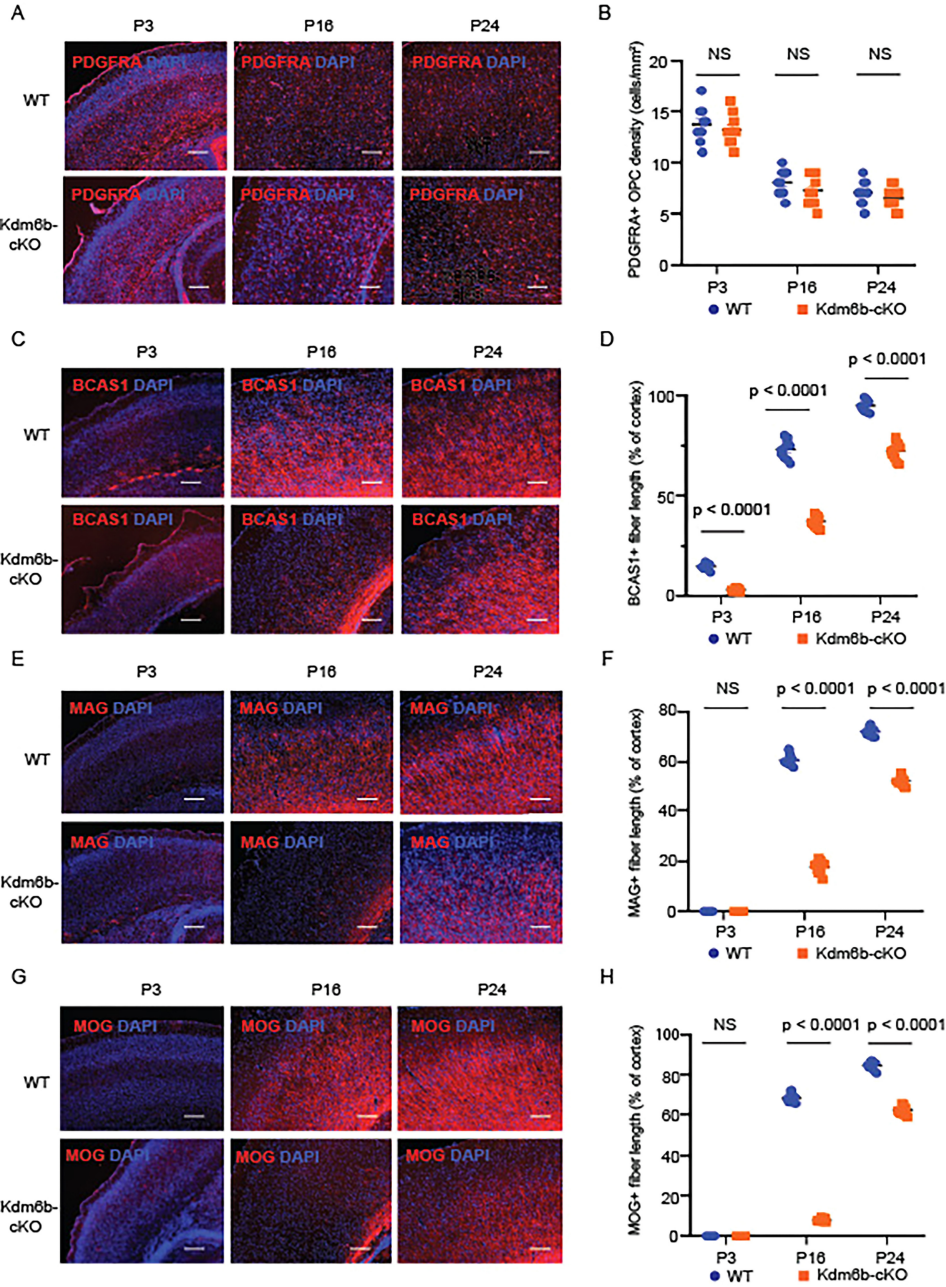
Loss of *Kdm6b* impairs postnatal oligodendrocyte maturation but not OPC density. **(A,B)** Representative cortical images from WT and Kdm6b-cKO mice at P3, P16, and P24 stained for PDGFRA (red) with DAPI (blue), and quantification of PDGFRA^+^ OPC density. **(C,D)** BCAS1 staining and quantification of BCAS1^+^ pre-myelinating oligodendrocyte (pre-OL) fiber length normalized to cortical length. **(E,F)** MAG staining and quantification of MAG^+^ mature oligodendrocyte (MOL) fiber length. **(G,H)** MOG staining and quantification of MOG^+^ mature oligodendrocyte (MOL) fiber length. Scale bars, 200 μm. Data are mean ± SEM. Each dot represents one biological replicate. Statistical significance is indicated in the panels; NS, not significant.

To determine whether the myelination defect in *Kdm6b*-cKO cortex reflects altered oligodendrocyte precursor cell (OPC) abundance or impaired oligodendrocyte maturation, we analyzed stage-specific markers in postnatal cortical sections. PDGFRA immunostaining showed that OPC density was similar between WT and *Kdm6b*-cKO mice at P3, P16, and P24 (Figures 3A,B). In contrast, BCAS1-positive pre-myelinating oligodendrocyte (pre-OL) fibers were significantly reduced in *Kdm6b*-cKO cortex at all three ages examined (Figures 3C,D). Similar to MBP, markers of mature oligodendrocytes (MOLs), including MAG and MOG, were unchanged at P3 but were significantly decreased at P16 and P24 in *Kdm6b*-cKO cortex (Figures 3E–H). To assess whether these changes were accompanied by altered neuronal abundance, we quantified NeuN-positive cells in the cortex at the same postnatal stages. NeuN^+^ neuronal density was comparable between WT and *Kdm6b*-cKO mice at P3, P16, and P24 (Supplementary Figure 3), indicating that the observed myelination defects were not associated with an overt change in postnatal cortical neuron density. Together, these findings indicate that *Kdm6b* loss does not detectably affect postnatal OPC or neuronal density, but is associated with impaired oligodendrocyte maturation during postnatal cortical development.

### Loss of *Kdm6b* impairs gene expression programs required for postnatal oligodendrocyte maturation

**FIGURE 4:**
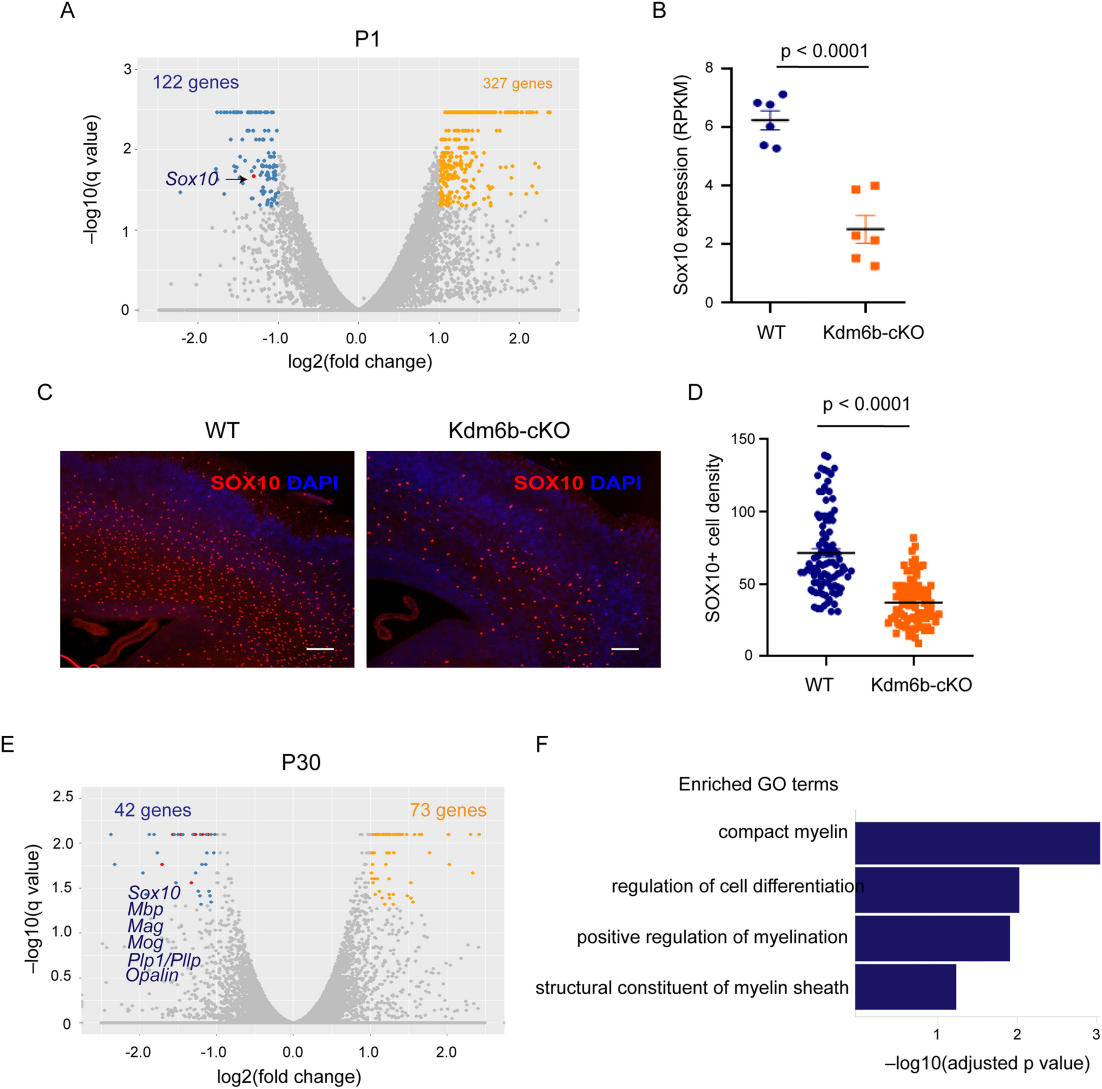
*Kdm6b* deletion suppresses *Sox10* expression and oligodendrocyte gene programs during cortical development. **(A)** Volcano plot of differentially expressed genes in P1 *Kdm6b*-cKO versus WT Emx1-lineage cortical cells. Blue and orange dots denote significantly downregulated and upregulated genes, respectively (|log2FC| > 1, FDR < 0.05). *Sox10* is highlighted among the downregulated genes. **(B)** RNA-seq quantification showing reduced *Sox10* expression in *Kdm6b*-cKO cortices at P1, expressed as reads per kilobase per million mapped reads (RPKM). Data are mean ± SEM; *p* < 0.0001, two-tailed Student’s *t*-test. **(C)** Representative immunofluorescence images of SOX10 (red) and DAPI (blue) in WT and *Kdm6b*-cKO cortices at P1. Scale bars, 200 μm. **(D)** Quantification of SOX10 + cell density in WT and *Kdm6b*-cKO cortices. Each dot represents an individual sampling region. Mean ± SEM; *p* < 0.0001. **(E)** Volcano plot of differentially expressed genes in P30 cortices, highlighting sustained downregulation of oligodendrocyte lineage genes including *Sox10, Mbp, Mag, Mog, Plp1*, and *Opalin* in *Kdm6b*-cKO mice. **(F)** Gene ontology (GO) enrichment analysis of downregulated genes in *Kdm6b*-cKO cortices at P30, revealing significant enrichment for myelin-related biological processes and oligodendrocyte differentiation programs.

To define the molecular mechanisms by which KDM6B regulates oligodendrocyte development and myelination, we performed RNA sequencing of Emx1^+^ lineage-derived cortical cells isolated at postnatal day 1 (P1) and postnatal day 30 (P30). For both WT and *Kdm6b*-cKO littermates, cortical nuclei were purified by FANS using the SUN1-GFP reporter prior to library preparation. At P1, differential expression analysis identified approximately 122 downregulated and 327 upregulated genes in *Kdm6b*-cKO cortices relative to WT controls (fold changes > 2, q < 0.05). Notably, *Sox10*, a master regulator of oligodendrocyte lineage progression, was among the most significantly downregulated genes (Figures 4A,B). Gene Ontology analysis of the P1 downregulated gene set showed enrichment for biological processes including cell-cell signaling, localization, regulation of nervous system process, and nervous system development (Supplementary Figure 4). Consistent with these transcriptomic changes, SOX10 immunostaining of P1 cortices revealed a marked reduction in SOX10 protein levels in Kdm6b-cKO mice (Figures 4C,D).

By P30, transcriptomic analysis identified 42 downregulated and 73 upregulated genes. Notably, Sox10 remained persistently suppressed, accompanied by broad downregulation of genes associated with mature oligodendrocyte function and myelin sheath formation, including Mbp, Mag, Mog, Plp1/Pllp, Opalin, and Cmtm5 (Figure 4E). Gene ontology analysis further revealed significant enrichment of downregulated genes in myelin-associated biological processes, including compact myelin formation, structural constituents of the myelin sheath, and positive regulation of myelination (Figure 4F).

Together, these dynamic transcriptional changes closely parallel the histological deficits observed in *Kdm6b*-cKO cortices and indicate that impaired cortical myelination results from disrupted oligodendrocyte lineage progression driven by loss of key regulatory programs, most notably *Sox10*.

### Loss of *Kdm6b* alters promoter-associated histone H3K27me3 levels at the *Sox10* locus

**FIGURE 5:**
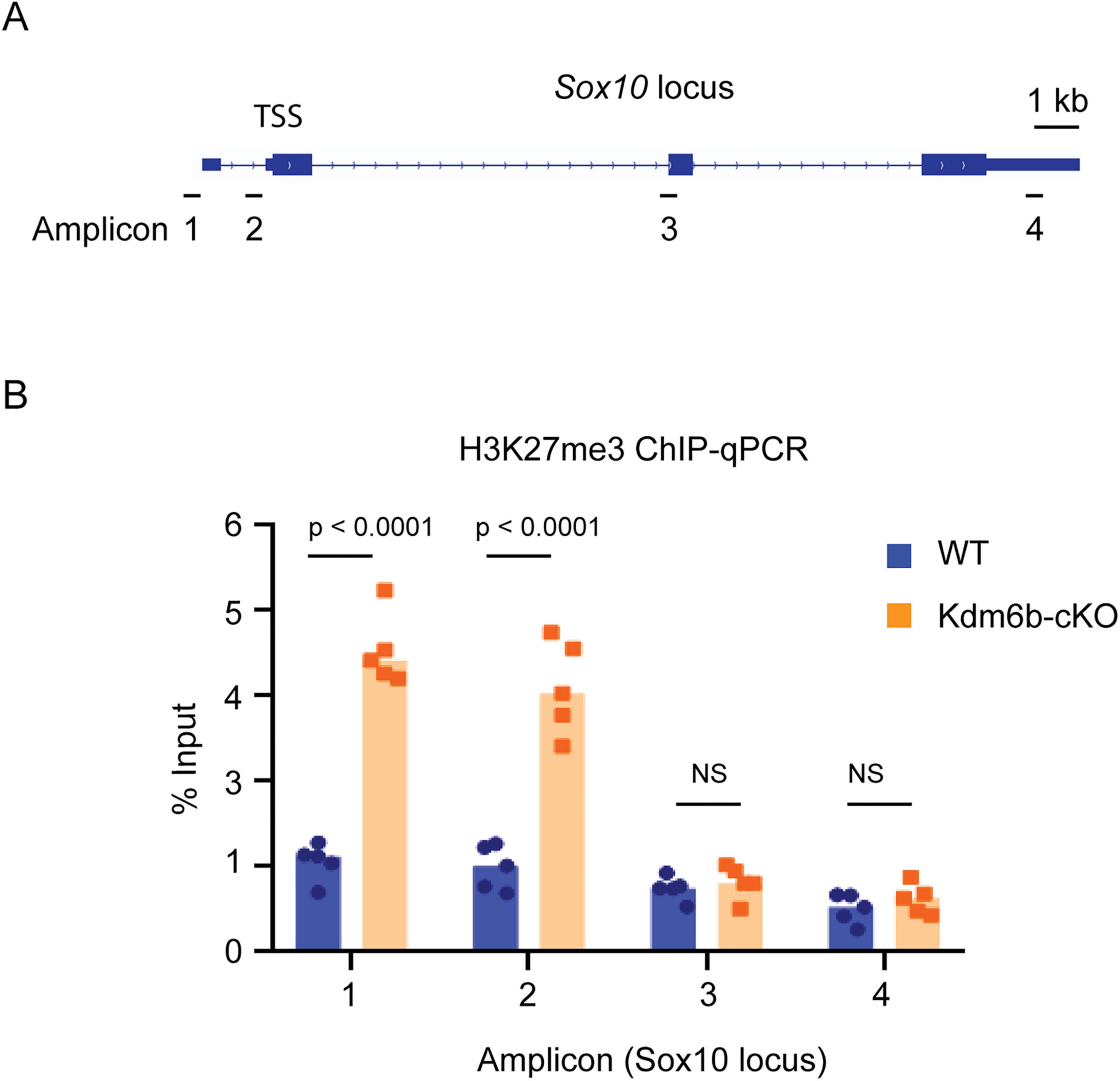
Loss of *Kdm6b* leads to accumulation of H3K27me3 at the Sox10 promoter. **(A)** Schematic of the *Sox10* locus with ChIP–qPCR amplicons positioned relative to the transcription start site (TSS). Scale bar, 1 kb. **(B)** H3K27me3 ChIP–qPCR analysis in FANS-isolated Emx1-lineage cortical cells from WT and *Kdm6b*-cKO mice. Enrichment (% input) is increased at promoter-proximal regions (amplicons 1–2) in Kdm6b-cKO samples, with no significant differences at distal regions (amplicons 3–4). Each dot represents one biological replicate; bars indicate mean ± SEM. Statistical significance is indicated in the panel; NS, not significant.

To assess the epigenetic consequences of KDM6B loss, we performed chromatin immunoprecipitation followed by quantitative PCR (ChIP–qPCR) on FANS-sorted Emx1-lineage cortical cells, focusing on histone H3K27me3, a repressive modification deposited by Polycomb repressive complex 2 (PRC2) and removed by KDM6B. Compared with WT controls, *Kdm6b*-cKO cells exhibited significantly increased H3K27me3 enrichment at promoter-proximal regions of genes that were transcriptionally downregulated upon *Kdm6b* deletion. Notably, H3K27me3 levels were markedly elevated at the *Sox10* locus at amplicons near the transcription start site, whereas more distal regions showed no significant changes (Figure 5). These data indicate that loss of *Kdm6b* is associated with accumulation of promoter-proximal H3K27me3 at the *Sox10* locus, consistent with reduced *Sox10* expression in Kdm6b-cKO cortices. Thus, impaired chromatin activation at the *Sox10* promoter is likely to contribute to the reduced expression of this key oligodendrocyte lineage regulator following *Kdm6b* deletion.

### Temporally controlled Sox10 expression partially rescues cortical myelination in *Kdm6b*-cKO mice

To test whether *Sox10* is a critical KDM6B target mediating postnatal oligodendrocyte development and cortical myelination, we attempted a rescue experiment by ectopically expressing *Sox10* in neural progenitors within the subventricular zone (SVZ) of the embryonic cortex using *in utero* electroporation (IUE) at E15.5. We initially employed a constitutive expression plasmid encoding Sox10-P2A-RFP, in which the self-cleaving P2A peptide generates separate SOX10 and RFP proteins, enabling fluorescent identification of transfected cells (Szymczak et al., 2004). A control plasmid expressing truncated LacZ-P2A-RFP was electroporated in parallel.

Constitutive *Sox10* overexpression at E15.5 profoundly disrupted radial migration, differentiation, and proliferation of cortical progenitors. Whereas LacZ-transfected neurons migrated normally and populated cortical layers II–III with typical neuronal morphology, *Sox10*-transfected progenitors largely failed to migrate and instead accumulated within the subventricular zone (SVZ). *Sox10*-expressing cells also exhibited reduced dispersion and prominent clustering near the ventricular region, consistent with impaired progenitor differentiation and proliferation (Supplementary Figure 5). Moreover, these cells lacked neuronal characteristics, as indicated by the absence of RFP-labeled callosal axons. Together, these findings demonstrate that premature *Sox10* expression in the embryonic cortex diverts progenitors toward non-neuronal fates that are incompatible with normal neuronal migration, differentiation, and proliferation during cortical development.

To circumvent premature fate switching, we implemented a tetracycline-inducible system to achieve temporal control of *Sox10* expression (Gossen and Bujard, 1992). TRE-(Sox10-P2A-RFP) or TRE-(LacZ-P2A-RFP) responder plasmids were co-electroporated with an EF1α-rtTA activator. Validation in HEK293T cells demonstrated robust doxycycline (Dox)-dependent induction, with markedly increased RFP fluorescence upon Dox treatment and minimal basal expression in its absence. Immunoblotting further confirmed an approximately fivefold increase in SOX10 protein levels compared with controls (Supplementary Figure 6).

**FIGURE 6:**
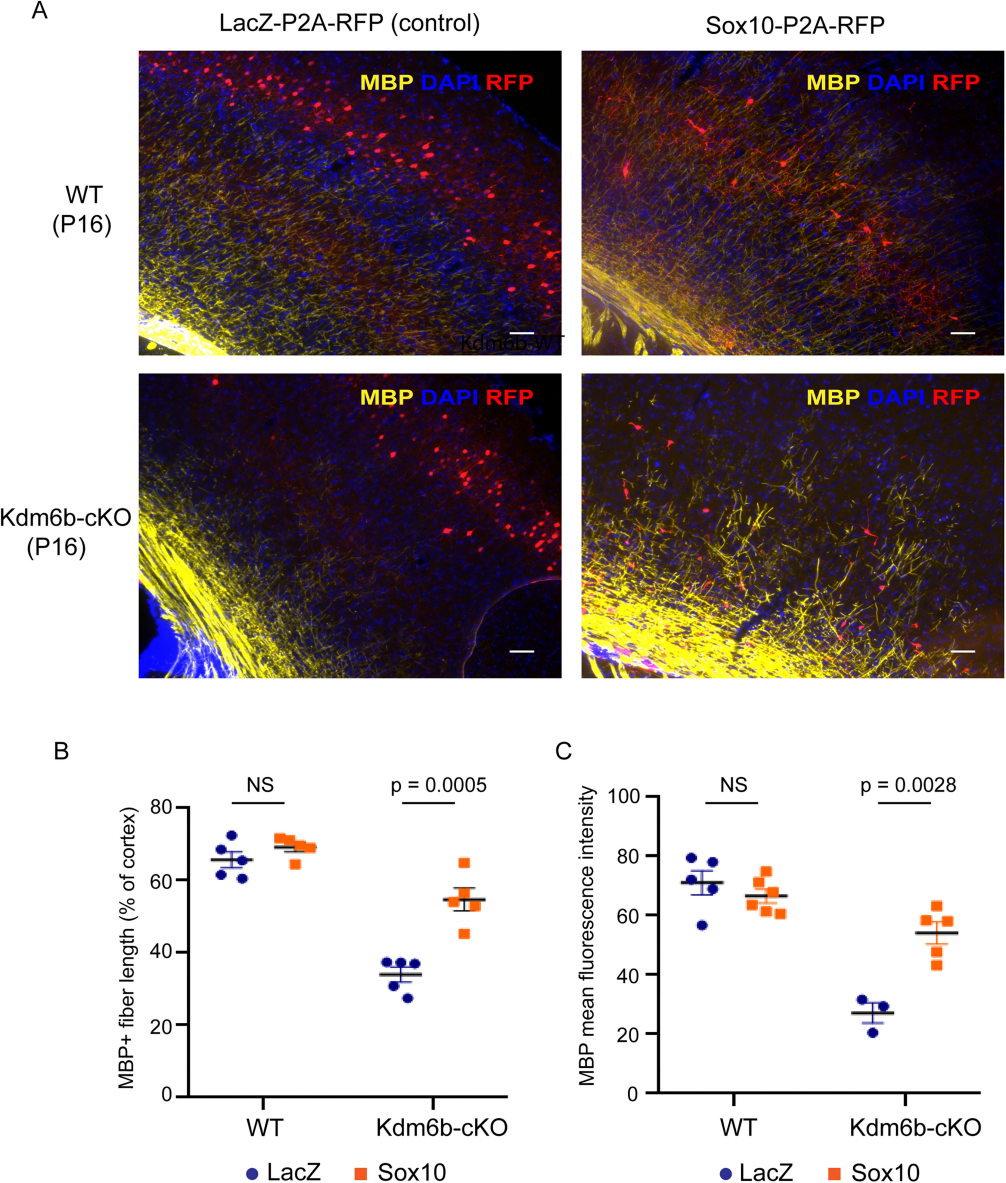
Temporally controlled *Sox10* expression partially rescues cortical myelination in Kdm6b-cKO mice. **(A)** Representative immunofluorescence images of cortical sections from Kdm6b-WT and Kdm6b-cKO mice at P16 following *in utero* electroporation of LacZ-P2A-RFP (control) or Sox10-P2A-RFP constructs. Sections were stained for myelin basic protein (MBP, yellow) and counterstained with DAPI (blue); RFP (red) marks electroporated cells. Scale bars, 200 μm. **(B)** Quantification of MBP-positive fiber length normalized to total cortical length. *Sox10* expression did not significantly alter myelination in WT cortex, but significantly increased MBP-positive fiber length in *Kdm6b*-cKO cortex compared with LacZ controls. **(C)** Quantification of mean MBP fluorescence intensity (MFI). *Sox10* overexpression did not significantly change MBP MFI in WT cortex, but significantly increased MBP MFI in *Kdm6b*-cKO cortex. Each dot represents one biological replicate; bars indicate mean ± SEM. Statistical significance is indicated in the panels; NS, not significant.

Using this inducible system, Dox was administered from E18.5 to P16 to activate transgene expression in postmitotic or late-stage cells. Under these conditions, LacZ-electroporated cells predominantly migrated to cortical layers II–III, whereas *Sox10*-electroporated cells accumulated mainly in layers IV–VI, with fewer cells reaching superficial layers (Supplementary Figure 7). Notably, inducible *Sox10* expression significantly increased cortical myelination in *Kdm6b*-cKO cortices at P16 compared with LacZ-transfected *Kdm6b*-cKO controls, although myelination did not fully reach wild-type levels (Figure 6). Together, these results indicate that temporally controlled *Sox10* expression partially rescues the myelination defect caused by *Kdm6b* loss, supporting SOX10 as a functionally important downstream effector of KDM6B in postnatal oligodendrocyte development.

## Discussion

Our study identifies KDM6B as a key epigenetic regulator of postnatal cortical myelination. Deletion of *Kdm6b* in Emx1^+^ dorsal telencephalic progenitors resulted in delayed cortical myelin accumulation during early postnatal development, accompanied by reduced MBP immunoreactivity and decreased maturation-associated oligodendrocyte markers. These anatomical defects were paralleled by molecular changes in Emx1-lineage cortical cells, including early downregulation of *Sox10* at P1 and later suppression of myelin-related genes such as *Mbp, Mag, Mog*, and *Pllp* by P30. In parallel, ChIP-qPCR demonstrated increased promoter-proximal H3K27me3 at the *Sox10* locus in *Kdm6b*-deficient cells. Together, these findings support a model in which KDM6B promotes postnatal oligodendrocyte maturation and cortical myelination, at least in part, by maintaining *Sox10* in a transcriptionally permissive chromatin state.

A central conclusion of this study is that SOX10 functions as the upstream master transcriptional regulator through which KDM6B exerts much of its effect on oligodendrocyte maturation. SOX10 occupies a pivotal position in the oligodendrocyte lineage hierarchy and is required for progression from precursor and pre-myelinating states into mature, myelinating oligodendrocytes (Stolt et al., 2002; Li et al., 2007; Pozniak et al., 2010). It acts upstream of multiple core myelin genes and differentiation effectors, thereby coordinating the broader transcriptional program necessary for oligodendrocyte maturation and myelin formation. In this context, the early and robust reduction of *Sox10* in *Kdm6b*-cKO cortices is especially significant: among the observed transcriptional changes, loss of this master regulator provides the most parsimonious explanation for the subsequent broad suppression of oligodendrocyte and myelin-associated genes. Thus, rather than acting primarily at the level of individual structural myelin genes, KDM6B appears to regulate oligodendrocyte maturation at a higher hierarchical level by preserving expression of the lineage-defining factor SOX10.

Several findings support this KDM6B→SOX10 model. First, *Sox10* was among the earliest and most strongly downregulated genes following *Kdm6b* deletion. Second, the *Sox10* locus gained repressive H3K27me3 in *Kdm6b*-cKO cortical cells. Third, temporally controlled *Sox10* induction partially rescued cortical myelination *in vivo*. Together, these results identify SOX10 as a functionally central downstream effector of KDM6B. The incomplete rescue, however, suggests that additional KDM6B-dependent pathways likely cooperate with SOX10 to achieve full oligodendrocyte maturation.

Our stage-specific histological analyses further refine this interpretation. *Kdm6b* loss did not measurably alter PDGFRA^+^ OPC density or NeuN^+^ neuronal density during the postnatal stages examined, arguing against a major defect in OPC maintenance or broad neuronal loss (Figure 3 and Supplementary Figure 3). In contrast, BCAS1^+^ pre-OLs were reduced early, whereas MBP^+^, MAG^+^, and MOG^+^ mature oligodendrocyte markers declined at later stages. However, these signals remained detectable and increased over time in *Kdm6b*-cKO cortex, indicating that the temporal progression of oligodendrocyte maturation and myelination is preserved, although attenuated. These findings therefore support a delay, rather than a complete block, in oligodendrocyte maturation and myelination, consistent with the known upstream role of SOX10 in driving this transition (Stolt et al., 2002; Li et al., 2007; Pozniak et al., 2010).

Our rescue experiments also highlight the importance of developmental timing. Constitutive *Sox10* overexpression at E15.5 disrupted cortical progenitor migration and differentiation, whereas delayed *Sox10* induction from late embryonic to postnatal stages partially restored myelination. These findings indicate that *Sox10* must be engaged within the proper developmental window and further support the idea that KDM6B promotes maturation by enabling timely activation of the SOX10-dependent program.

An important feature of this study is its Emx1-lineage focus. Although embryonic cortical oligodendrocytes originate from ventral sources, dorsally derived Emx1^+^ progenitors contribute substantially to postnatal oligodendrogenesis (Gorski et al., 2002; Tripathi et al., 2011; Crawford et al., 2016). Our data show that KDM6B is required within this dorsal lineage to maintain *Sox10* expression and support downstream myelin gene activation, identifying KDM6B as a regulator of dorsally derived oligodendrocyte maturation.

These findings may have clinical relevance. KDM6B variants are increasingly linked to neurodevelopmental disorders (Stessman et al., 2017; Stolerman et al., 2019; Satterstrom et al., 2020), and our data suggest that impaired postnatal oligodendrocyte maturation may be one downstream consequence. In this framework, SOX10-centered transcriptional networks emerge as particularly important mediators of KDM6B function.

Several limitations warrant consideration. First, restriction to the Emx1 lineage complicates quantitative attribution across all oligodendrocyte sources; future fate-mapping and cell type-specific genetic studies will refine mechanistic resolution. Second, *Sox10* induction did not fully normalize myelination, which may reflect suboptimal timing or dosage, incomplete coverage of the SOX10 regulome, or contributions from *Sox10*-independent KDM6B targets. Third, our chromatin analysis focused on promoter-proximal H3K27me3 at the *Sox10* locus; broader genome-wide chromatin approaches, such as CUT&Tag or ChIP-seq, will be important in future studies to define the full set of KDM6B-regulated loci and the broader chromatin landscape underlying oligodendrocyte maturation, including potential enhancer-level and higher-order chromatin mechanisms.

In summary, our findings support a model in which KDM6B acts upstream of the master oligodendrocyte regulator SOX10 to promote postnatal cortical myelination. By limiting repressive H3K27me3 at the *Sox10* locus, KDM6B helps sustain *Sox10* expression and thereby enables the downstream transcriptional program required for oligodendrocyte maturation and myelin formation. Loss of KDM6B disrupts this upstream control point, leading to reduced SOX10, impaired oligodendrocyte maturation, and delayed postnatal myelination.

## Data Availability

The datasets presented in this study can be found in online repositories. The names of the repository/repositories and accession number(s) are provided below: Gene Expression Omnibus (GEO), GSE328562.

## Footnotes

1
https://biit.cs.ut.ee/gprofiler/gost
