# Supplementary material for: Histone demethylase KDM6B promotes postnatal oligodendrocyte maturation and cortical myelination
Source: Front Cell Neurosci. 2026 May 19;20:1832157. doi: 10.3389/fncel.2026.1832157 (PMC13225964; doi:10.3389/fncel.2026.1832157)
Supplement: Supplementary file 1 [file Presentation_1.pdf]

.

Supplementary Information

Histone Demethylase KDM6B Promotes Postnatal Oligodendrocyte Maturation and Cortical Myelination

Ruth Lambries[1,3], Zihan Shen[1,3], George I Mias[1, 2], Jin He[1*]

[1]Department of Biochemistry and Molecular Biology, College of Natural Science, Michigan State University, East Lansing, MI, United States

[2]Institute for Quantitative Health Science and Engineering, Michigan State University, East Lansing, MI, United States

Correspondence to: hejin1@msu.edu

This file includes:

Figures S1 to S7
Table S1

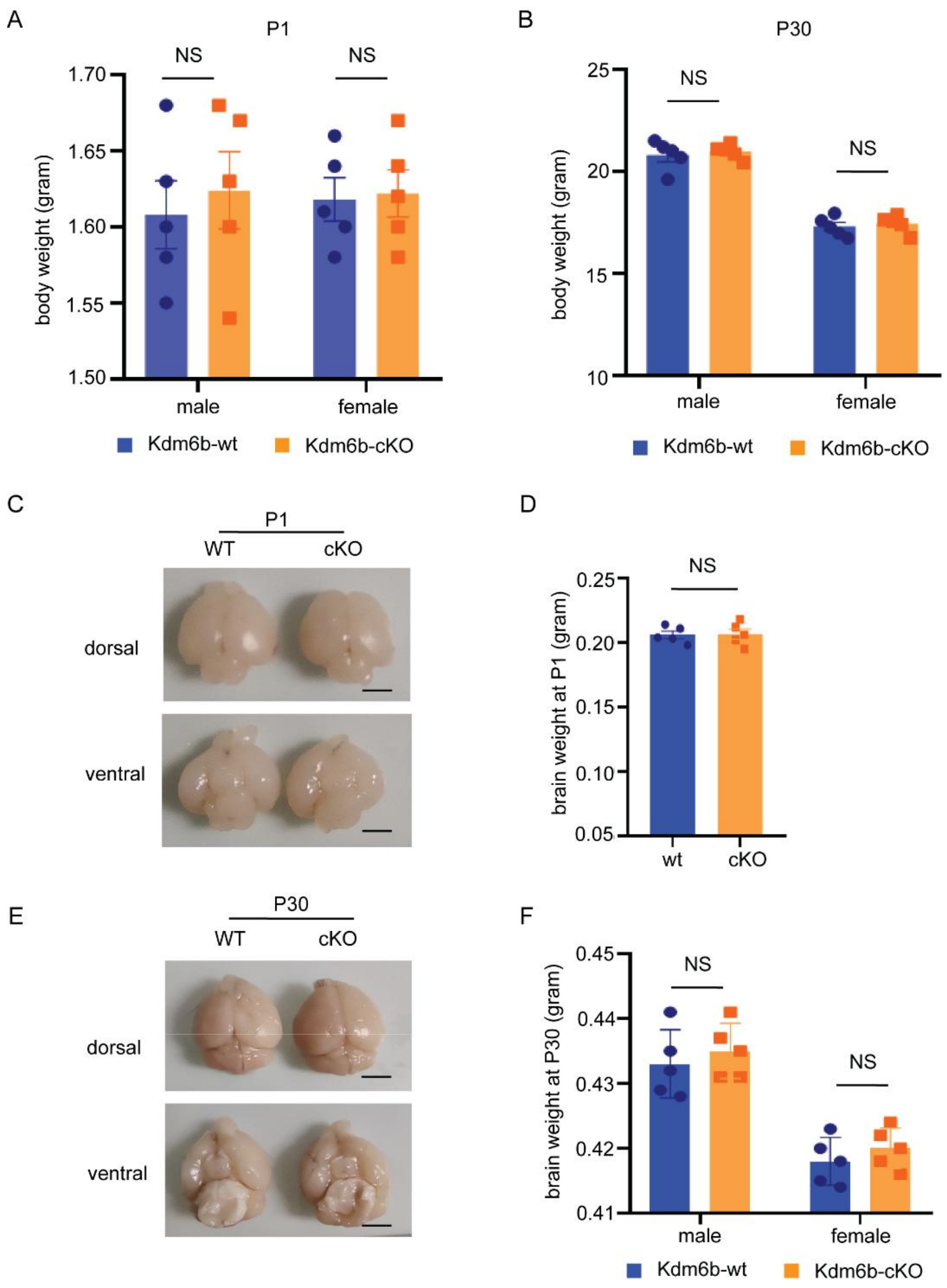

Figure S1. Body and brain weights are unchanged in Kdm6b-cKO mice at P1 and P30.
(A, B) Body weight measurements of wild-type (WT) and Kdm6b conditional knockout (cKO) mice at postnatal day 1 (P1) and postnatal day 30 (P30), shown separately for males and females. No significant differences were detected between genotypes at either age. NS, not significant.
(C, E) Representative dorsal and ventral views of whole brains from WT and cKO mice at P1 and P30. Scale bars, 2 mm. NS, not significant.
(D, F) Quantification of brain weight at P1 and P30. No significant differences were observed between WT and cKO mice. Scale bars, 5 mm. NS, not significant.

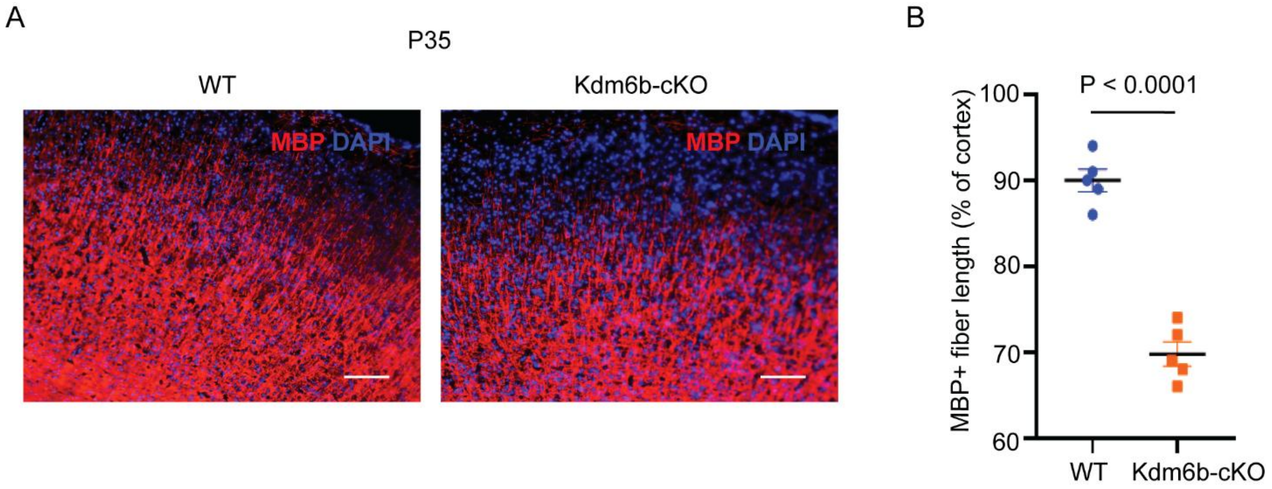

Figure S2. MBP-positive fiber length remains reduced in *Kdm6b*-cKO cortex at P35.
(A) Representative cortical sections from WT and *Kdm6b*-cKO mice at P35 immunostained for MBP (red) with DAPI counterstain (blue).
(B) Quantification of MBP-positive fiber length normalized to cortical length. MBP-positive fiber length remained significantly reduced in *Kdm6b*-cKO cortex compared with WT at P35. Each dot represents one biological replicate; bars indicate mean ± SEM. Scale bars, 200 µm. Statistical significance is indicated in the panel.

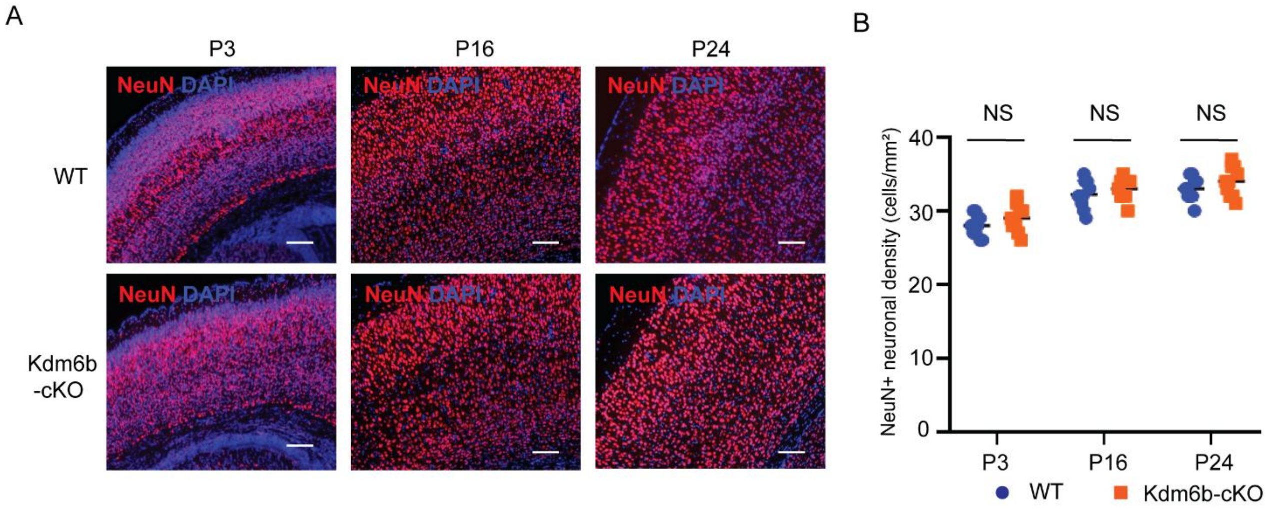

Figure S3. Neuronal density is not altered in *Kdm6b*-cKO cortex.
(A) Representative cortical sections from WT and *Kdm6b*-cKO mice at P3, P16, and P24 immunostained for NeuN (red) with DAPI counterstain (blue).
(B) Quantification of $NeuN^+$ neuronal density ($cells/mm^2$) in the cortex at the indicated ages. No significant differences were observed between WT and *Kdm6b*-cKO mice at any time point. Each dot represents one biological replicate; bars indicate mean ± SEM. Statistical significance is indicated in the panels; NS, not significant.

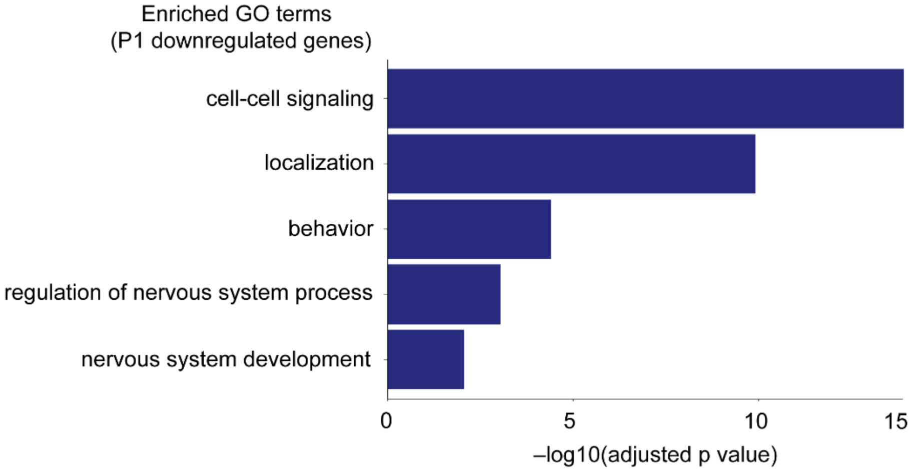

Figure S4. Gene Ontology analysis of downregulated genes in *Kdm6b*-cKO Emx1-lineage cortical cells at P1. Bar plot showing the top enriched GO biological process terms associated with genes downregulated in *Kdm6b*-cKO samples relative to WT controls at postnatal day 1 (P1). Bars represent enrichment significance as –log10(adjusted p value).

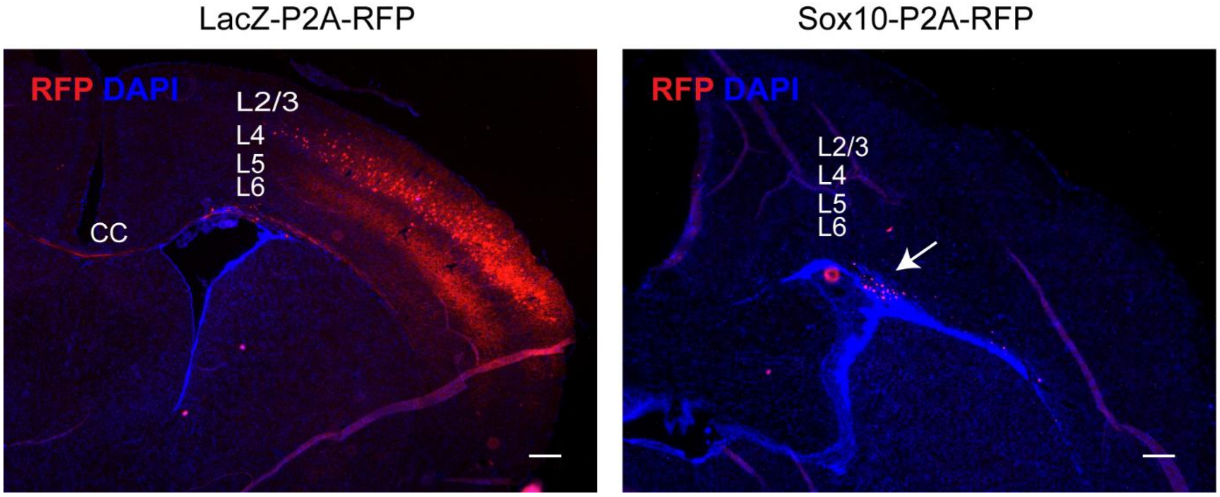

Figure S5. Early *Sox10* expression disrupts cortical laminar organization.
Representative cortical sections following in utero electroporation of LacZ-P2A-RFP (control) or Sox10-P2A-RFP constructs at E15.5. RFP (red) marks electroporated cells and DAPI (blue) labels nuclei. In control cortex, RFP^{+} cells are appropriately distributed across cortical layers, whereas Sox10 overexpression results in abnormal cell positioning and disrupted laminar organization (arrow). Cortical layers (L2/3–L6) and corpus callosum (CC) are indicated. Scale bars, 200 µm.

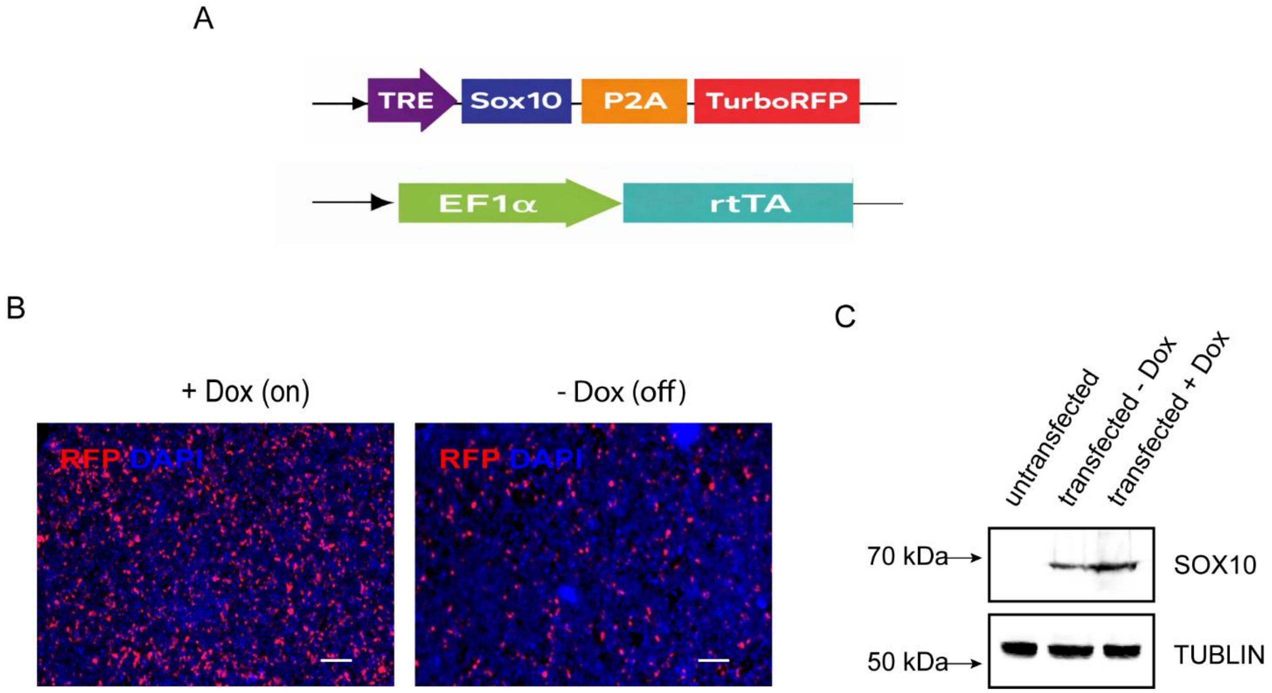

Figure S6. Validation of doxycycline-inducible *Sox10* expression system.
(A) Schematic of the Tet-On system used for inducible *Sox10* expression, consisting of a TRE-driven Sox10-P2A-TurboRFP cassette and an EF1α-driven rtTA construct.
(B) Representative images of cultured cells transfected with Sox10-P2A-RFP constructs in the presence (+Dox) or absence (–Dox) of doxycycline. RFP (red) marks induced expression and DAPI (blue) labels nuclei. Robust RFP expression is observed with doxycycline treatment, with minimal basal expression in the absence of doxycycline.
(C) Immunoblot analysis of SOX10 protein expression in untransfected cells and transfected cells cultured with or without doxycycline. SOX10 protein is strongly induced by doxycycline, whereas only minimal leaky expression is detected in the absence of doxycycline. Tubulin serves as a loading control.

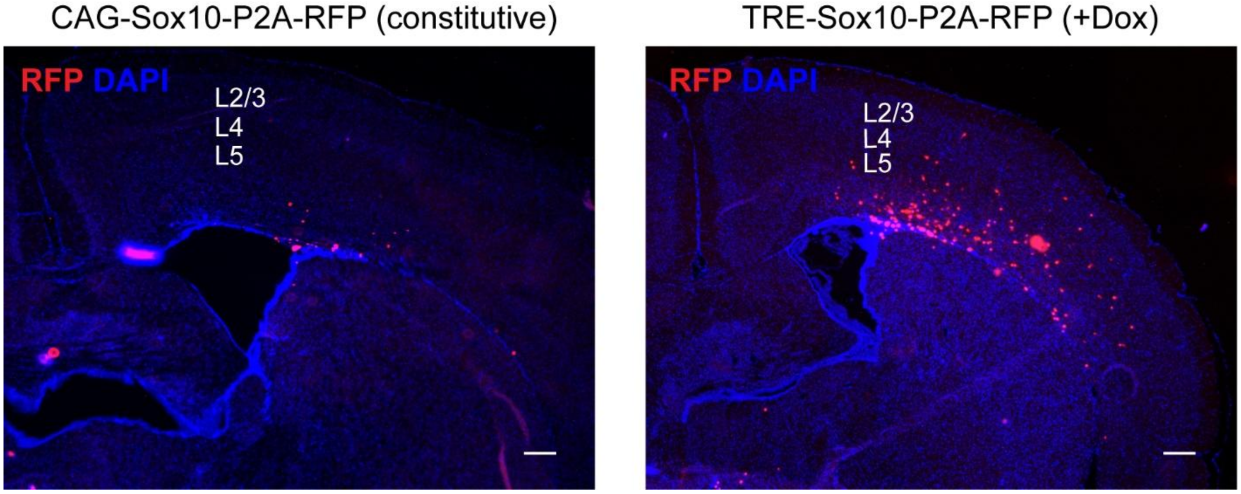

Figure S7. Temporal control of Sox10 expression mitigates disruption of cortical organization. Representative cortical sections following in utero electroporation of CAG-Sox10-P2A-RFP **(**constitutive) or TRE-Sox10-P2A-RFP (doxycycline-inducible) constructs. RFP (red) marks electroporated cells and DAPI (blue) labels nuclei. Constitutive Sox10 expression results in abnormal distribution of RFP^+ cells across cortical layers, whereas inducible Sox10 expression shows a broader radial distribution than constitutive Sox10 expression. Cortical layers (L2/3–L5) are indicated. Scale bars, 200 µm.

Supplementary Table 1. Sequences of all primers used in this study

Name	Sequence (5'-3')	Purpose
GAPDH-F	gcagtggcaaagtggagatt	qRT-PCR
GAPDH-R	gaatttgccgtgagtggagt	qRT-PCR
Kdm6b-F	agaggaaccagacagcactac	qRT-PCR
Kdm6b-R	cttccacctcttggcatca	qRT-PCR
ChIP_Sox10_amp1_F	ctgactgtgccacctgtatc	ChIP-qPCR
ChIP_Sox10_amp1_R	ctggactcagcttgggttt	ChIP-qPCR
ChIP_Sox10_amp2_F	aacgccttcatggtgtgg	ChIP-qPCR
ChIP_Sox10_amp2_R	cagagcttgcctagtgtcttg	ChIP-qPCR
ChIP_Sox10_amp3_F	ctgacccttgtagctccattt	ChIP-qPCR
ChIP_Sox10_amp3_R	cagcctaagatggttgtgatct	ChIP-qPCR
ChIP_Sox10_amp4_F	gcctgtctcttggtctcttac	ChIP-qPCR
ChIP_Sox10_amp4_R	ggaaagcgcctaaggaatct	ChIP-qPCR
